# Consolidated bioprocessing of *Populus* using *Clostridium* (*Ruminiclostridium*) *thermocellum*: a case study on the impact of lignin composition and structure

**DOI:** 10.1186/s13068-016-0445-x

**Published:** 2016-02-04

**Authors:** Alexandru Dumitrache, Hannah Akinosho, Miguel Rodriguez, Xianzhi Meng, Chang Geun Yoo, Jace Natzke, Nancy L. Engle, Robert W. Sykes, Timothy J. Tschaplinski, Wellington Muchero, Arthur J. Ragauskas, Brian H. Davison, Steven D. Brown

**Affiliations:** Biosciences Division, Oak Ridge National Laboratory, Oak Ridge, TN 37831 USA; BioEnergy Sciences Center, Bioscience Division, Oak Ridge National Laboratory, Oak Ridge, TN 37830 USA; Renewable Bioproducts Institute, Georgia Institute of Technology, Atlanta, GA 30332 USA; UT-ORNL Joint Institute for Biological Sciences, Oak Ridge National Laboratory, Oak Ridge, TN 37831 USA; School of Chemistry and Biochemistry, Georgia Institute of Technology, Atlanta, GA 30332 USA; National Renewable Energy Laboratory, US Department of Energy, Golden, CO 80401 USA; Department of Chemical and Biomolecular Engineering, Department of Forestry, Wildlife, and Fisheries, University of Tennessee, Knoxville, TN 37996 USA

**Keywords:** Lignin, Syringyl, Guaiacyl, S/G ratio, Consolidated bioprocessing, *Populus*, *Clostridium thermocellum*, Molecular weight

## Abstract

**Background:**

Higher ratios of syringyl-to-guaiacyl (S/G) lignin components of *Populus* were shown to improve sugar release by enzymatic hydrolysis using commercial blends. Cellulolytic microbes are often robust biomass hydrolyzers and may offer cost advantages; however, it is unknown whether their activity can also be significantly influenced by the ratio of different monolignol types in *Populus* biomass. Hydrolysis and fermentation of autoclaved, but otherwise not pretreated *Populus trichocarpa* by *Clostridium thermocellum* ATCC 27405 was compared using feedstocks that had similar carbohydrate and total lignin contents but differed in S/G ratios.

**Results:**

*Populus* with an S/G ratio of 2.1 was converted more rapidly and to a greater extent compared to similar biomass that had a ratio of 1.2. For either microbes or commercial enzymes, an approximate 50 % relative difference in total solids solubilization was measured for both biomasses, which suggests that the differences and limitations in the microbial breakdown of lignocellulose may be largely from the enzymatic hydrolytic process. Surprisingly, the reduction in glucan content per gram solid in the residual microbially processed biomass was similar (17–18 %) irrespective of S/G ratio, pointing to a similar mechanism of solubilization that proceeded at different rates. Fermentation metabolome testing did not reveal the release of known biomass-derived alcohol and aldehyde inhibitors that could explain observed differences in microbial hydrolytic activity. Biomass-derived *p*-hydroxybenzoic acid was up to nine-fold higher in low S/G ratio biomass fermentations, but was not found to be inhibitory in subsequent test fermentations. Cellulose crystallinity and degree of polymerization did not vary between *Populus* lines and had minor changes after fermentation. However, lignin molecular weights and cellulose accessibility determined by Simons’ staining were positively correlated to the S/G content.

**Conclusions:**

Higher S/G ratios in *Populus* biomass lead to longer and more linear lignin chains and greater access to surface cellulosic content by microbe-bound enzymatic complexes. Substrate access limitation is suggested as a primary bottleneck in solubilization of minimally processed *Populus*, which has important implications for microbial deconstruction of lignocellulose biomass. Our findings will allow others to examine different *Populus* lines and to test if similar observations are possible for other plant species.

**Electronic supplementary material:**

The online version of this article (doi:10.1186/s13068-016-0445-x) contains supplementary material, which is available to authorized users.

## Background

Recent advancements in lignocellulosic biofuel strategies have shown increased adoption and improved conversion of dedicated bioenergy feedstocks. For example, consolidated bioprocessing (CBP) growth on very high loads of minimally processed switchgrass has been described for *Caldicellulosiruptor bescii* [1]. Yeast-based simultaneous saccharification and fermentation (SSF) and consolidated bioprocessing with *Clostridium thermocellum* have shown improved bioconversion performance for switchgrass with reduced lignin content [2]. Bioconversion performances for *Saccharomyces cerevisiae* SSF and several CBP approaches have been assessed for switchgrass (*Panicum virgatum*) under different conditions [3]. *C. thermocellum* has one of the highest rates for cellulose utilization [4]. Metabolic engineering has generated strains that produce 70 % of theoretical ethanol yield on Avicel and ethanol titers up to 73.4 mM, although further engineering is required [5].

**Fig. 1:**
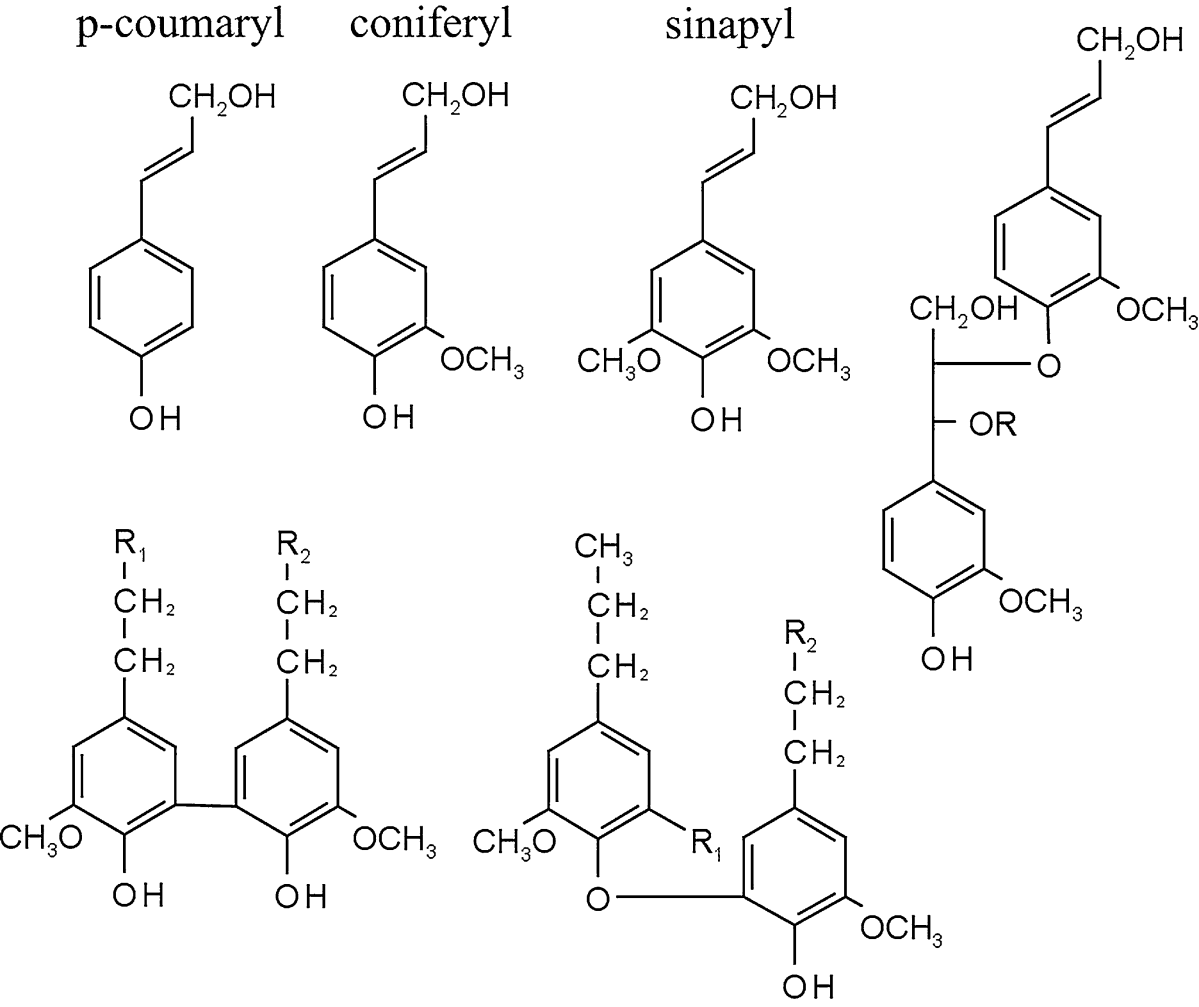
*Top* The three primary monolignols (from *left* to *right*) that form H, G, and S lignin units (respectively) and the most common linkage in the lignin polymer, the β-O-4 ether bond (*right-most*); *Bottom* highly resistant 5-5 (*left*) and β-5 (*right*) lignin linkages facilitated by the G unit

*Populus* is a fast-growing woody bioenergy feedstock investigated for utilization in large scale bioconversion to alcohols [6, 7]. Its inherent recalcitrance to enzymatic and microbial deconstruction is one of the largest impediments to large scale, economically feasible biofuel production. Understanding *Populus* properties responsible for its resistance to degradation will aid in the generation of low recalcitrance plants. Lignin is an important component of lignocellulosic biomass, which is thought to act as a physical barrier toward the accessible surface of carbohydrates and adsorb and inactivate cellulases to restrict enzymatic hydrolysis [8]. Lignin is a branched heterogeneous polymer that makes up 16–28 % of the content of undomesticated natural variants of *Populus trichocarpa* [9]. When incorporated into lignin, the primary monolignols (Fig. 1) form three units: *p*-hydroxyphenylpropane (H), syringyl (S), and guaiacyl (G). S and G are the predominant units in the hardwood lignin backbone and vary in an S/G ratio between 1.0 and 3.0 [9]. Lignin is most commonly linked through a labile arylglycerol-β-aryl ether (*β*-*O*-*4*) bond [10].

Compositionally, these lignin units differ by the absence of the methoxy group in position five on the benzene ring of the G unit, which results in the potential creation of two additional highly resistant bonds [11], the 5-5 and the β-5 linkages (Fig. 1). This fundamental difference has been theorized to prompt differences in biomass degradability when the S and G lignin content varies: G-rich lignin could promote thinner cell walls that are easier to degrade [12], or it may lead to denser polymer crosslinking with increased resistance to degradation [13]; S-rich lignin may form predominantly linear shorter chains with thermoplastic properties more favorable to degradation, particularly by hydrothermal pretreatments [9].

Despite the emerging hypotheses, current literature does not hold a unified mechanistic understanding on the role of lignin in the biological breakdown of lignocellulosic biomass. Still, there is general agreement that lignification significantly inhibits deconstruction, in particular, a strong negative correlation is seen between total lignin content and sugar release by free-enzyme hydrolysis [14, 15]. However, literature data do not show a generalized association between lignin composition and cellulose degradability in lignocellulose. Several reports found no discernable correlation between lignin S/G content and saccharification levels in alfalfa transgenics [15], synthetic cell wall complexes [16], and untreated *Arabidopsis thaliana* mutants [17]. A high S/G ratio was found to adversely affect xylose release by acid hydrolysis in *Populus* [13], the enzymatic solubilization of maize [18], and transgenic *Populus* degradation by wood-decay fungi [19]. At the same time, high S/G was found to improve the saccharification of pretreated *A. thaliana* mutants [17], the efficiency of Kraft pulping [20], and enzymatic sugar release in undomesticated *Populus* [9]. A challenge in comparing these published results is that many other properties beyond S/G ratio may also vary in these studies. These examples demonstrate that lignin S and G variations can be neutral or relevant depending on plant species, transgenic modifications, biomass pretreatments, and the choice of degradation agent or method.

For undomesticated natural variants of *Populus trichocarpa*, the biomass tested in the current study, Studer and collaborators [9] found an S/G ratio above 2.0 to generally improve enzymatic hydrolysis, while the worst nine out of 47 performers had either very low S/G ratios (below 1.2) or very high total lignin content (above 27.8 %). Cellulose properties, such as the degree of polymerization [21] and crystallinity [22], have also been of interest for characterization of lignocellulose recalcitrance and were investigated in this study in relation to lignin.

In this study, for the first time, we address whether lignin S/G variations have an impact on the consolidated bioprocessing of *Populus* biomass by a model cellulolytic organism, *Clostridium thermocellum* ATCC 27405. We evaluate the bioconversion performance of *Populus* individuals with similar average total lignin values and high or low S/G compositions to determine whether microbes have differential access to sugars, whether potential inhibitor release was linked to lignin composition, and whether the abundance of S and G-units was responsible for changes in biomass structural properties before and after fermentation (i.e., lignin and cellulose molecular weights, cellulose crystallinity, and degree of polymerization).

## Results

### Initial microbial bioconversion screening of *Populus* natural variants

**Fig. 2:**
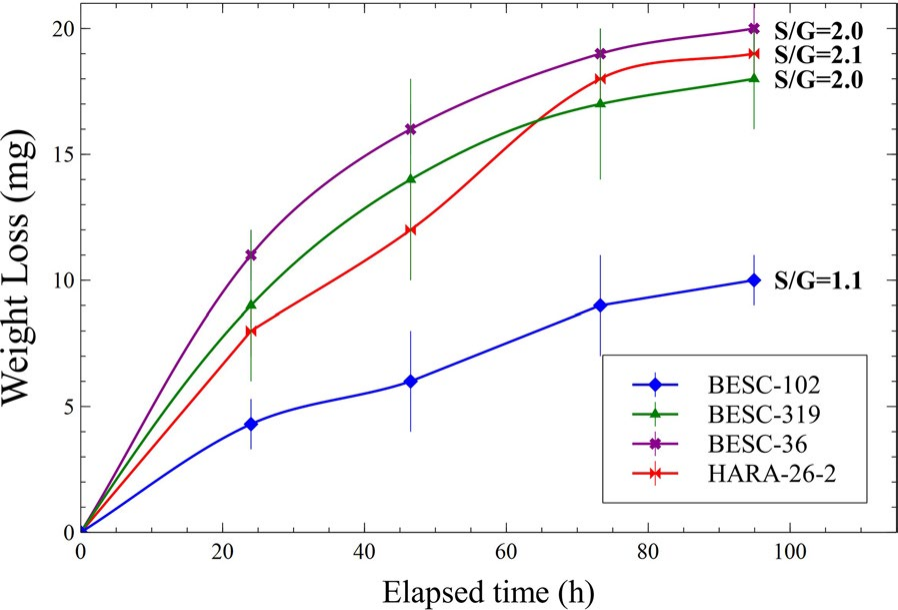
Bioconversion screening through time-course-weight loss measurements in batch fermentations with *C. thermocellum* ATCC 27405 at 5 g/L (dry basis) initial biomass loadings. Mean values and standard deviations are shown for triplicate fermentations

*Populus* natural variants were screened and selected on the basis of average and similar total lignin (~24 %) content. A subsection was assayed for primary carbohydrate content (i.e., glucose, xylose, galactose, arabinose, and mannose) and the lignin S/G ratio. These selected *Populus* had very similar sugar contents (Additional file 1: Figure A.1). Three with average S/G ratios (~2.1) and one with the lowest possible S/G ratio (~1.2) were chosen for bioconversion performance assessment. Microbial CBP screening of these *Populus* individuals revealed a very similar performance in samples with equal S/G ratios, and a significantly lower conversion of the *Populus* with very low S/G content (Fig. 2). The results are consistent with reported solubilization of undomesticated *Populus* with commercial enzyme mixtures [9].

To investigate what was responsible for the large discrepancy in the degradability of *Populus* with seemingly comparable sugar and total lignin content, but with a two-fold change in S/G content, two individuals were selected for further comparative analyses and will be referred to as the “low S/G” (natural variant BESC-102) and the “high S/G” (natural variant HARA-26-2) phenotypes.

### Consolidated bioprocessing (CBP): microbial hydrolysis and fermentation of low and high S/G *Populus* phenotypes

**Fig. 3:**
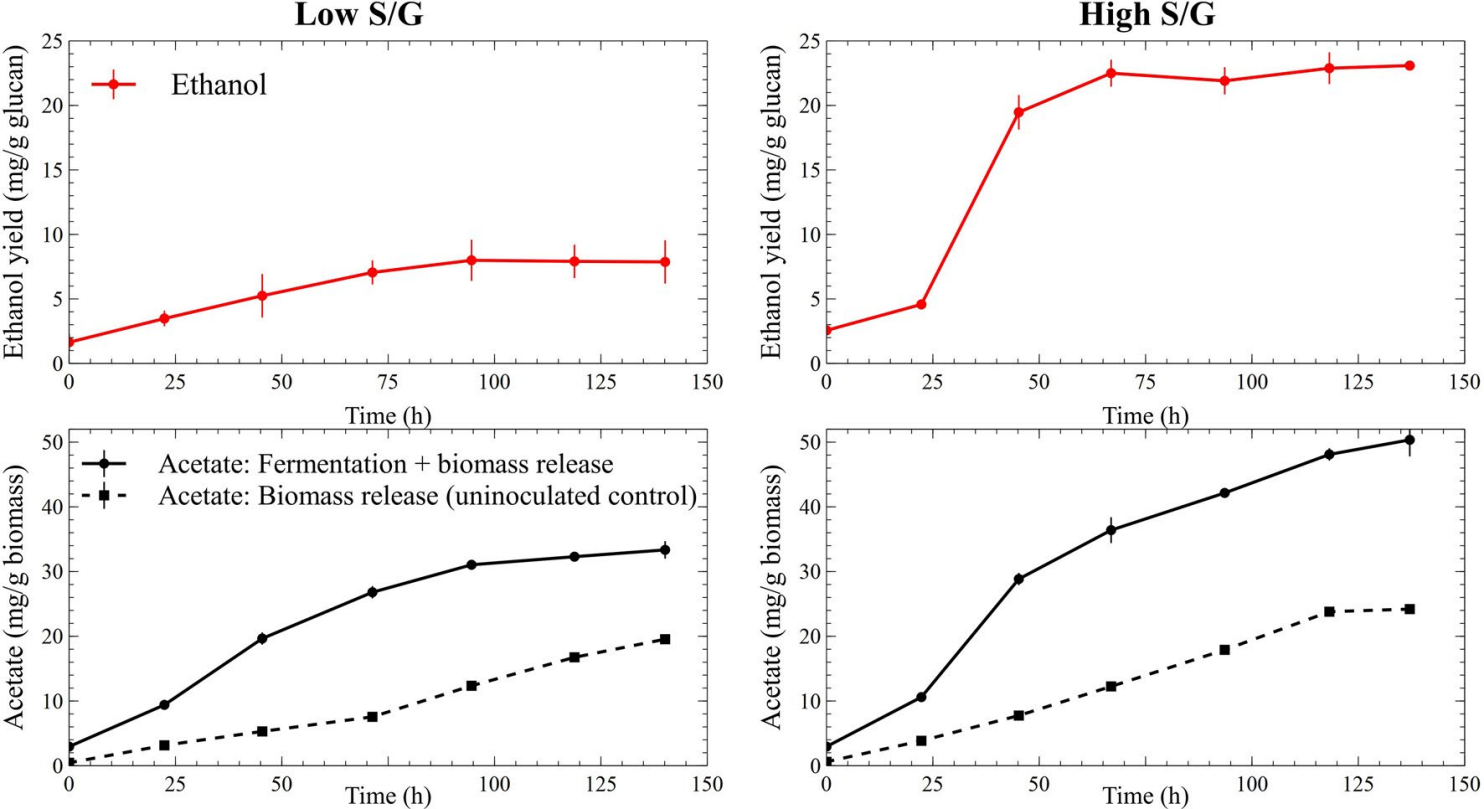
A 2.9-fold ethanol yield increase in the consolidated bioprocessing of high S/G *Populus* biomass over the low S/G variant (*top*); biomass-derived acetate (*below*, *dotted line*) measured in un-inoculated bioreactor controls and total acetate measured in CBP bioreactors (*below*, *solid line*)—fermentative acetate is approximately the difference between these two series. Mean values and standard deviations are shown for triplicate fermentations

**Fig. 4:**
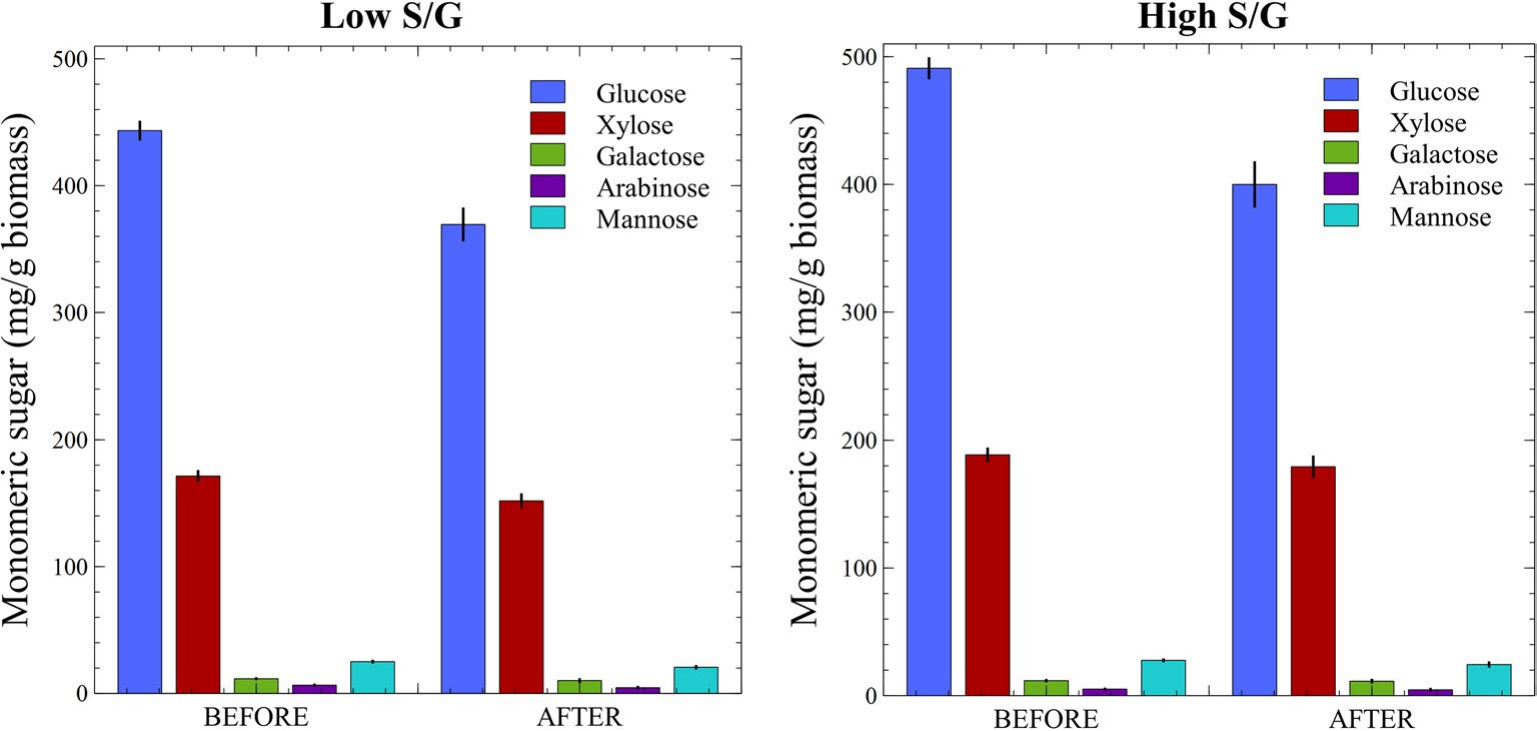
Sugar content per gram solid in low S/G (*left*) and high S/G (*right*) *Populus* before and after consolidated bioprocessing. Mean values and standard deviations are shown for solids of triplicate fermentations

Microbial hydrolysis and fermentation of 20 g/L biomass samples (approximately 8–8.8 g/L glucan) in pH-controlled bioreactors showed total solid solubilization of 12 % in low S/G and 20 % in high S/G *Populus* (i.e., a 50 % relative difference—calculated as absolute difference normalized to average solubilization for both lines). Ethanol yield (mg/g glucan) was 2.9-fold higher for the high S/G *Populus*, although fermentations of low S/G samples had their metabolic output shifted more toward acetate production—reported here as normalized to total biomass (Fig. 3). Subtracting the end-point biomass-derived acetate of control (uninoculated) samples from the total acetate in fermentation samples, the rough acetate to ethanol ratio was calculated to be 2.9:1 and 5.4:1 for the high S/G and the low S/G biomass conversions, respectively. Xylose release was proportional to total solid solubilization in both cases (data not shown). Control fermentations with 10 g/L glucan (Avicel) showed near-complete solubilization in less than 24 h (Additional file 1: Figure A.2). Compositional analysis of primary sugars in the two *Populus* phenotypes, before and after microbial bioconversion revealed an equal 15 % reduction (g/g solid, dry basis) in the measured total carbohydrates. Glucose recorded the largest reductions in absolute values; however, with very similar relative changes (per gram solid), at 17 and 18 % lower post-fermentation glucan content in the low S/G and the high S/G phenotypes, respectively (Fig. 4).

### Separate hydrolysis and fermentation (SHF): free-enzyme hydrolysis and yeast fermentation of low and high S/G *Populus* phenotypes

**Fig. 5:**
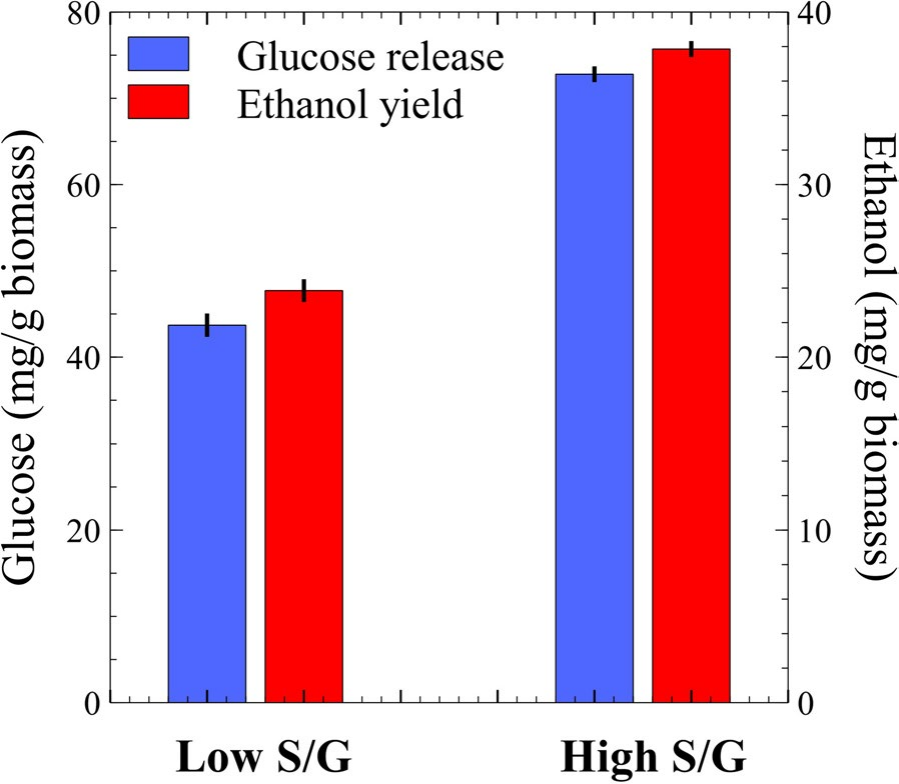
Glucose release and subsequent yeast fermentation to ethanol in separate hydrolysis and fermentation (SHF) of the low and high S/G *Populus*. Mean values and standard deviations are shown for triplicate fermentations

To test whether the large differences observed under CBP conversion were largely at the hydrolysis level, the two *Populus* phenotypes were also processed with a mixture of commercial cellulases, hemicellulases, and a *β*-glucosidase at 50 °C. A 52 % relative difference in glucose release was measured in favor of the high S/G phenotype. Xylose release measured a similar 60 % relative difference (data not shown), and the subsequent yeast fermentation of solubilized sugars had similar conversion efficiency to ethanol (i.e., ethanol yield/glucose release, under 5 % relative difference) (Fig. 5), indicating no biomass-derived adverse effect on the yeast metabolism.

### GC–MS metabolome analysis of CBP bioconversions for identification of potential inhibitor release

In order to test whether biomass-derived inhibitors were contributors to differences in biomass solubilization during microbial hydrolysis, extracellular bioreactor metabolome samples were analyzed at time zero, 71, and 140 h. Unsurprisingly, guaiacyl lignans were released in higher proportion from the low S/G *Populus*, while syringyl lignans were released in higher proportion from the high S/G biomass (Table 1). However, the very low detected concentrations were below what was considered inhibitory. The most common biomass-derived inhibitors (e.g., lignin precursors, HMF, furfural) were also not detected in the fermentation medium of either feedstock.

**Table 1:** Time-course GC–MS metabolome analysis of CBP bioconversions; aqueous concentrations (mg/L) and the fold change (low S/G to high S/G) of potential biomass-derived inhibitors

Metabolite	0 h			71 h			140 h		
	Low S/G	High S/G	Fold change	Low S/G	High S/G	Fold change	Low S/G	High S/G	Fold change
*p*-Hydroxybenzoic acid	1.28	0.14	*9.33*	4.54	0.56	*8.08*	6.82	1.09	*6.26*
4-Hydroxyphenethyl alcohol	0.02	0.01	*1.50*	0.26	0.77	*0.34*	0.46	1.73	*0.27*
11.64 347	0.38	0.16	*2.37*	4.62	0.97	*4.78*	8.23	1.74	*4.72*
11.00 218 100	2.13	0.55	*3.89*	5.16	1.28	*4.04*	4.20	1.30	*3.23*
10.92 218 100 277	3.38	1.08	*3.14*	9.32	3.71	*2.51*	7.73	2.41	*3.21*
14.90 Guaiacyl lignan	1.33	0.36	*3.73*	1.15	0.36	*3.19*	0.72	0.28	*2.61*
14.96 Guaiacyl lignan	2.30	0.68	*3.36*	2.27	0.81	*2.80*	1.67	0.70	*2.37*
Guaiacylglycerol	0.09	0.04	*2.17*	0.10	0.05	*2.20*	0.09	0.05	*2.00*
15.52 Syringyl lignan	0.09	0.17	*0.55*	0.12	0.25	*0.49*	0.13	0.28	*0.46*
15.44 Syringyl lignan	0.05	0.10	*0.55*	0.05	0.10	*0.47*	0.04	0.10	*0.38*
Syringylglycerol	0.09	0.09	1.04	0.13	0.12	1.09	0.12	0.13	0.92

Italicized values: higher in the Low S/G poplar bioconversion; Italicized underlined values: higher in the High S/G poplar bioconversion

Only metabolites released with greater than two-fold difference between the low and high S/G phenotypes are shown (with the exception of syringyl glycerol). Numerical labels for unidentified metabolites are denoted by retention time and key mass-to-charge (*m/z*) ratios

**Fig. 6:**
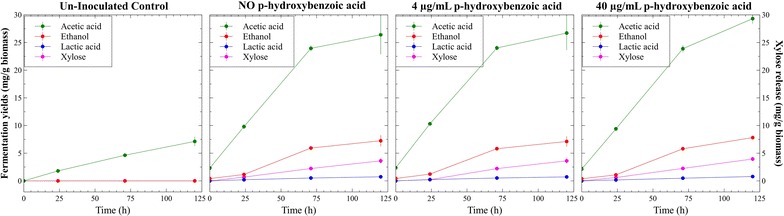
CBP fermentations of the high S/G *Populus* phenotype with additions of potential inhibitor *p*-hydroxybenzoic acid showed no significant effect on metabolic output. The compound was added to the culture medium in quantities that produced final concentrations close to onefold and tenfold the concentrations found in the low S/G cultures. Mean values and standard deviations are shown for triplicate fermentations

Among the identified metabolites, *p*-hydroxybenzoic acid was released in higher concentrations (up to 6.8 µg/mL) and also showed the most pronounced fold-differences between the two *Populus* phenotypes. In order to test the potential inhibitory effect of *p*-hydroxybenzoic acid, it was added to fermentations of the high S/G *Populus* at two concentrations and was found to have no measurable impact on fermentation performance (Fig. 6).

### Biomass characterization: cellulose degree of polymerization, crystallinity, and lignin molecular weight

**Fig. 7:**
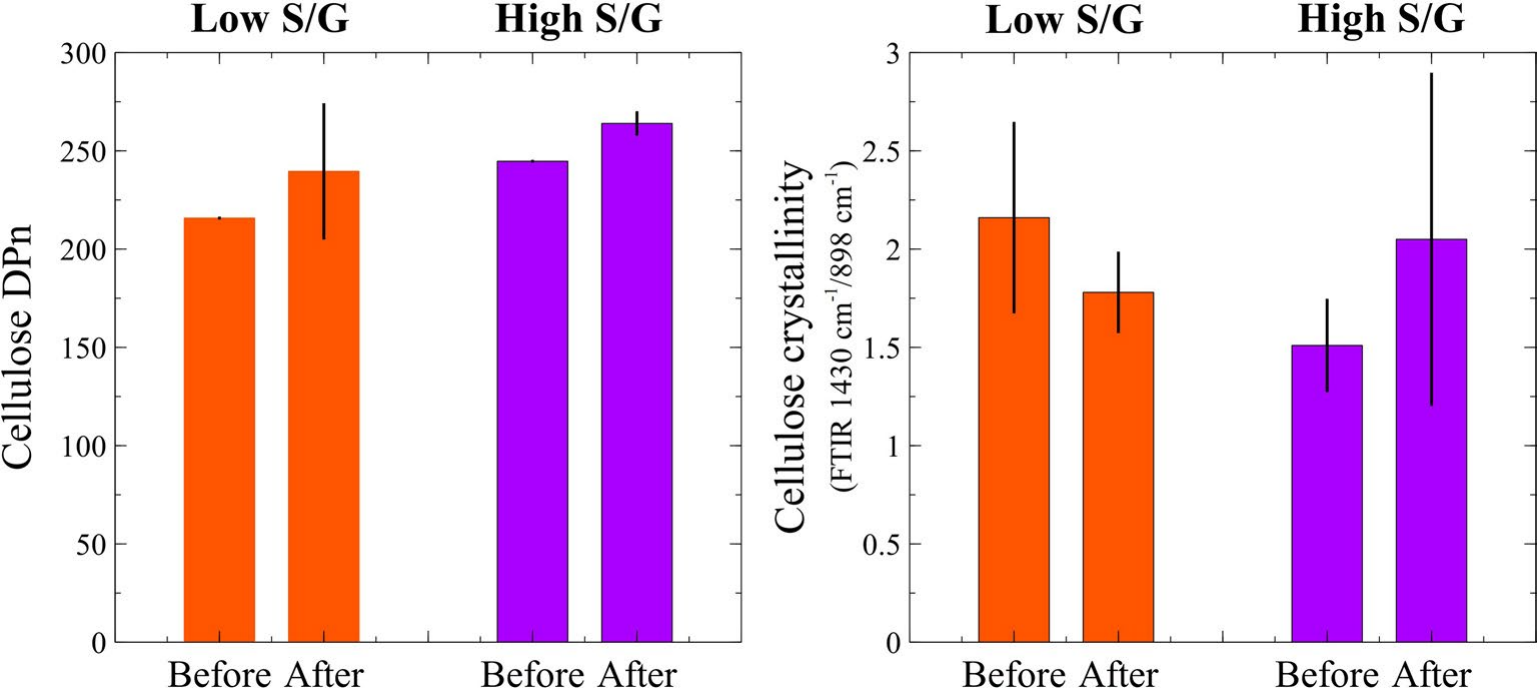
Cellulose number-average degree of polymerization (DP_n_) for “low S/G” and “high S/G” *Populus*, before and after CBP bioconversion. In both phenotypes, cellulose chain length increased slightly after fermentation; however, differences in cellulose properties between the two feedstock are not particularly significant (*left*); the ratio of crystalline to amorphous cellulose showed no significant differences between the two *Populus* phenotypes (*right*)

Cellulose was isolated from the low and high S/G biomasses, before and after CBP fermentation. The number-average and the weight-average degree of polymerization (DP_n_ and DP_w_, respectively) for cellulose were calculated from molecular weight determinations obtained by gel permeation chromatography (GPC) (Fig. 7). The high ratio S/G *Populus* had marginally longer cellulose chains (i.e., DP_n_); however, the difference became statistically not significant after bioconversion (*p* = 0.41). The degree of polymerization increased in both feedstocks following microbial hydrolysis by roughly 19–24 units. Polydispersity measurements provide indications of the broadness of molecular weight distributions for polymers. Polydispersity (DP_w_/DP_n_) calculations indicated that the low S/G *Populus* had a wider distribution of cellulose molecular weights (19.1 for low S/G and 16.6 for high S/G *Populus*), which did not change to a great extent after fermentation. Cellulose crystallinity was assessed using ATR-FTIR (attenuated total reflectance-Fourier transform infrared) to determine its relationship to S/G ratio. Peaks at 1430 and 898 cm^−1^ in the FTIR spectrum are sensitive to changes in crystalline and amorphous cellulose content [23, 24] and thus, the ratio of these peaks provides for comparisons of cellulose crystallinities (Fig. 7). The low S/G *Populus* displayed an initially higher crystallinity ratio (*p* = 0.22) compared to the starting high S/G *Populus*, which decreased following CBP conversion (*p* = 0.29). The high S/G biomass started with a lower initial crystallinity ratio that increased after microbial conversion (p = 0.46). However, differences were not statistically significant at 95 % confidence and the wider variations may be credited to heterogeneity in the small subsamples used in this analysis.

The crystallinity indices as determined by NMR ranged between 49.6 and 54.0 %, which are typical values for poplar [25]. The crystallinity indices indicate minor differences between the samples. The low S/G ratio biomass experienced a 4 % decrease in crystallinity, whereas the high S/G ratio biomass experienced a negligible change before and after CBP. The CrIs determined from NMR were in good agreement with the ATR-FTIR absorbance ratios for the samples with the exception of the unfermented high S/G poplar. Hydrogen bonding with water broadens peaks in the IR spectrum, which may account for the difference observed.

**Fig. 8:**
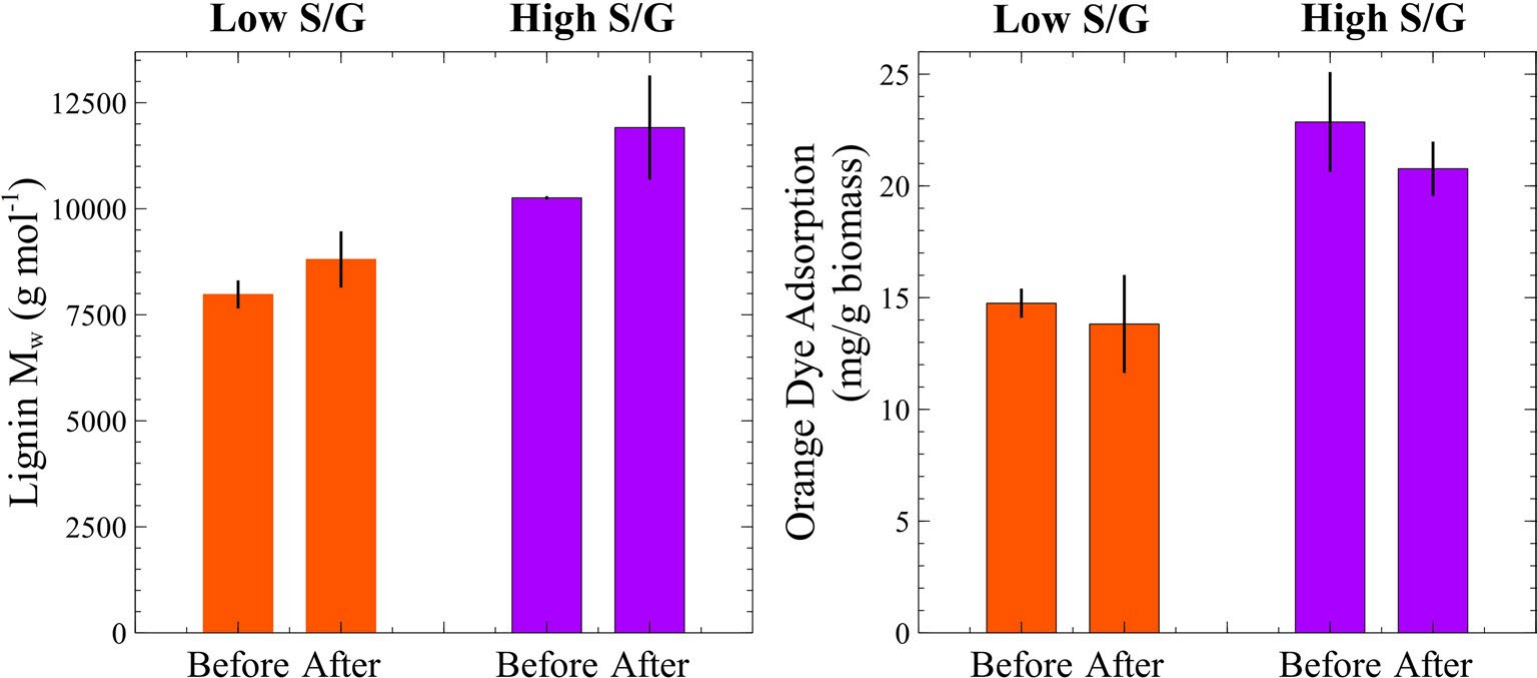
Lignin molecular weights (*M*_w_) are notably larger in the high S/G *Populus* wood before and after CBP conversion (*left*); the direct orange dye adsorption, determined by modified Simons’ staining, revealed better cellulose accessibility in the high S/G *Populus* phenotypes before and after fermentation (*right*)

Lignin was also isolated from the two *Populus* phenotypes before and after CBP conversion, and its molecular weight (in g/mol) was determined by GPC. The results pointed to the most meaningful difference between these *Populus* individuals (Fig. 8), where the high S/G variant had significantly larger lignin molecular weights than the low S/G biomass (*p* < 0.01) in both the initial and the CBP-processed samples. Post-fermentation biomass had modest lignin *M*_w_ increases in both phenotypes (although with low statistical significance, *p* = 0.13 and *p* = 0.11). The polydispersity indices were not different between the S/G variants (i.e., 2.2) and marginally increased to 2.4 only in the high S/G *Populus* after CBP conversion.

### Simons’ staining: cellulose accessibility measurements

Simons’ staining utilizes mixtures of a small blue dye (~1 nm molecular diameter) and a larger orange dye (~5–36 nm) to characterize cellulose surface porosity [26]. In this study, we report the orange dye adsorption values (Fig. 8), because the orange dye has a higher affinity to cellulose and is thought to provide a good estimation of enzymatic accessibility to cellulose surface area due to the dye’s molecular weight similarity to cellulases and its high binding affinity [27]. The high S/G *Populus* had better orange dye adsorption than the low S/G *Populus* (*p* = 0.04); and in both cases, these values decreased by small, although statistically not significant, amounts after CBP fermentation (*p* = 0.60 for low S/G and *p* = 0.24 for high S/G samples).

## Discussion

Based on literature investigations, to our knowledge, the impact of the syringyl and guaiacyl content in various natural and synthetic substrates has been studied almost exclusively using free-enzyme systems. Microbial lignocellulose conversion quite often relies on adherent microbes that have improved access to substrates and in general show better hydrolytic performance than their purified cellulase preparations [28]. The objective of this study was to expand our comprehension of lignocellulose recalcitrance to microbial solubilization and in particular when the content of syringyl units is nearly equal to (low S/G ratio) or two-fold higher (high S/G ratio) than the guaiacyl monolignol content. The research presented here investigated a small sample size of a single-plant species, and represents a case study intended to identify key biomass properties that impact solubilization of biomass by microbes and purified enzymes. Further validation requires larger sample sets and/or multiple plant species.

Our initial screening of natural *Populus* variants showed *C. thermocellum* bioconversions could be just as sensitive to biomass compositional properties, and in particular with respect to lignin, as hydrolysis with commercial enzyme preparations [9]. Microbial hydrolysis of biomass in bioreactors showed a relative 50 % improvement in solubilization for the unpretreated high S/G *Populus* compared the low S/G biomass. *C. thermocellum* bioconversion was comparable to the 52 % relative difference in the glucose release from the hydrolysis of these plant lines with commercial enzyme blends, which indicate that the performance differentiator in microbial conversion is also likely at the level of enzymatic activity, rather than due to disparities in microbial growth. Surprisingly, both *Populus* samples had an average 15 % relative reduction in total measured carbohydrates following microbial conversion, while glucan alone was reduced by 17–18 % per gram of post-fermentation recovered solid, indicating that glucose extraction, or mining, from the lignocellulosic matrix did not occur preferentially in either *Populus* biomass. The limited solubilization in all tests is typical for the minimally processed (milled, washed, and autoclaved) woody biomass that was used to accentuate lignin differences without gross alterations [29, 30]. Considering the large relative difference in total solid solubilization between these phenotypes, it is reasonable to conclude that microbial hydrolysis occurred in a similar mechanistic pattern, where the high S/G *Populus* was deconstructed at a faster rate. The evidence denotes less restricted enzyme access to the carbohydrates of the biomass with higher S/G lignin ratio; unfortunately, literature provides little corroboration on the actual mechanisms of complex lignocellulose breakdown by cellulolytic organisms.

A bias toward acetate production was observed in the fermentation of the low S/G *Populus*, and it is thought to be the consequence of lower carbon availability; a phenomenon also observed during the growth of this organism on pure cellulose substrates [31]. Previous reports cataloged the release of *Populus*-derived inhibitors against *C. thermocellum* after dilute acid pretreatment [32]; however, our fermentation metabolome analysis performed under the same GC-MS technique, did not reveal significant accumulation of these products which may be a result of using different biomass materials and/or different preparation methods. A previously unidentified component, *p*-hydroxybenzoic acid, was observed in this study but also found non-inhibitory when it alone was added in high levels. It remains uncertain if *p*-hydroxybenzoic acid is associated with lignin or hemicelluloses. Under the circumstances, differences in the activity of cell-bound enzymes could not be linked to the presence of aqueous inhibitors.

It was also important to compare the properties of cellulose and lignin in the two *Populus* samples that had been postulated in the literature to have a role in biomass solubilization, and to observe how these properties changed due to microbial activity. A lower cellulose degree of polymerization (DP) has been typically associated with greater availability of cellulose chain ends and therefore improved hydrolysis [21]. Our *Populus* phenotypes, exhibited cellulose of similar average chain length which marginally increased after fermentation, a phenomenon also observed in the utilization of birchwood by *C. thermocellum* [33]. In general, lower cellulose crystallinity improves hydrolysis with free-enzymes [22]. While cellulose accessibility can be affected by crystallinity, other attributes such as lignin/hemicellulose contents and distribution, porosity can play roles in cellulose digestibility [34], and the influence of crystallinity on consolidated bioprocessing with cellulolytic bacteria is less clear. Cellulose hydrolysis by *C. thermocellum* or its cellulosome has been proposed to occur at equal rates on crystalline and amorphous cellulose [33] and in other instances to occur rapidly on microcrystalline cellulose [35, 36]. In the current study, cellulose crystallinity in the two *Populus* tested was not affected by lignin composition, and did not significantly change under CBP treatment. Crystallinity is not considered a performance differentiator in the solubilization of these *Populus* phenotypes.

Lignin molecular weight was the strongest indication of structural differences in the compared biomasses; where a low S/G ratio resulted in greatly reduced lignin molecular weights. These results deviate from previous postulations that syringyl-rich lignins possess more fragmented structures and smaller molecular weights [9]. GPC separates polymers based on molecular size and assumes the polymer of interest to have spherical conformation in solution [37]; therefore, variations in calculated molecular weights may be attributed to differences in polymer chain size or conformation (i.e., a given polymer may appear low weight if it was highly branched, or high weight if it was straight chain). Hypothetically, structural disparities between lignins with low and high S/G ratios may stem from the radical polymerization of lignin. Coniferyl alcohol polymerization, which incorporates G-units into lignin, generates a more condensed lignin structure due to increased proportions of carbon–carbon linkages [38]. The compact structure of lignin isolated from low S/G *Populus* may manifest itself as a lower molecular weight, whereas the less compact structure of the high S/G phenotype may produce higher relative molecular weights.

Simon stains suggests the potential for greater enzyme accessibility to cellulose in samples with higher syringyl content [26], which points to higher surface porosity in the range of 5–36 nm and above. Although this may explain differences in enzymatic solubilization with free cellulases, this porosity range may not equally aid microbes with much larger footprints (i.e., micron size) which utilize complex, cell-bound cellulosome clusters. However, it remains to be determined whether the correlation between lignin composition and molecular size and the porosity of cellulose fibrils extends to future observations.

## Conclusions

While microbial hydrolysis of unpretreated or minimally pretreated lignocellulosic substrates is considered more efficient than with cell-free enzyme mixtures, we found biomass properties to have equal impact on their capacity to solubilize celluloses and hemicelluloses. In particular, solubilization was greatly improved on the *Populus* phenotype with a higher ratio of syringyl-to-guaiacyl lignin. The results found that the two significant properties of *Populus* tested in this study were cellulose accessibility and lignin molecular weight, both positively correlated to the S/G ratio. We suggest that higher syringyl content leads to larger linear lignin chains, which present less interference for carbohydrate breakdown by free-enzymes and microbes.

## Methods

### Microbial strains and culture media

*Clostridium thermocellum* strain ATCC 27405 was maintained in 20 % glycerol stock cultures at −80 °C. For batch bottle cultures, the strain was grown in defined MTC medium which was prepared anaerobically by mixing independently sterilized solutions. All medium components are reported here at final complete medium concentrations, per liter of ultrapure water. Initially, solution A (2.5 or 10 g Avicel PH-101, 0.001 g Resazurin) was sterilized by autoclave. Solution D (1 g magnesium chloride hexahydrate, 0.2 g calcium chloride dehydrate, 0.1 g ferrous chloride tetrahydrate, 1 g l-cysteine hydrochloride monohydrate) was also prepared fresh for each set of experiments and sterilized by autoclave. The remaining solutions, sterilized by filtration on 0.2 µm filters, contained the following: solution B + M (2 g potassium citrate, 1.25 g citric acid monohydrate, 1 g sodium sulfate dibasic, 1 g potassium phosphate monobasic, 2.5 g sodium bicarbonate, 5 g MOPS hemisodium salt), solution C (1.5 g ammonium chloride, 2 g urea), solution E (20 mg pyridoxamine dihydrochloride, 1 mg riboflavin, 1 mg nicotinamide, 0.5 mg lipoic acid, 4 mg 4-aminobenzoic acid, 4 mg D-biotin, 0.025 mg folic acid, 2 mg cyanocobalamin, 0.2 mg thiamine hydrochloride), and solution F (0.5 mg manganese chloride tetrahydrate, 0.5 mg cobalt chloride hexahydrate, 0.2 mg zinc sulfate heptahydrate, 0.05 mg copper sulfate pentahydrate, 0.05 boric acid, 0.05 mg sodium molybdenum oxide dihydrate, 0.05 mg nickel chloride hexahydrate, 10 mg citric acid monohydrate). Filter sterilized solutions (B + M, C, E, F) can be prepared as concentrated stocks (25×, 50×, 50×, and 1000×, respectively). Medium composition for pH-controlled batch bioreactor cultures was similar, with the replacement in solution A of Avicel with *Populus* biomass (20 g/L on a dry basis) and the omission of MOPS hemisodium salt from solution B + M.

*Saccharomyces cerevisiae* D5A (ATCC 200062) was maintained on YPD (10 g/L yeast extract, 20 g/L peptone, and 10 g/L glucose, 20 g/L agar) plates, and the same broth medium was used for overnight growth.

### *Populus* biomass preparation and compositional analysis

Four-year old tree of *Populus* genotypes BESC-102 and HARA-26-2 was harvested from a field site in Clatskanie, OR (46°6′11′′N 123°12′13′′W). Details of field establishment, growth conditions, and post-harvest handling were previously described by Muchero et al. [39].

*Populus* stem sections were milled (Mini Wiley Mill, Thomas Scientific, Swedesboro, NJ, USA), size selected using a 20-mesh screen (i.e., maximum 0.84 mm particle size), and autoclaved at 121 °C for 20 min at 20 g/L concentration. The biomass was then washed with 1 L of ultrapure water for every 10 g solids. Washed biomass was then used in the preparation of solution A in batch bottle and bioreactor media where it was loaded wet, but on a dry basis, based on previously determined dry-to-wet weight ratios and autoclaved for a second time during the sterilization of solution A in medium preparation steps. Carbohydrate content in washed biomass did not change significantly (Additional file 1: Figure A.3); total lignin content decreased slightly (possibly due to washout of very small wood particulates) while S/G ratios did not change (Additional file 1: Figure A.4). The purpose of autoclave-washing was to reduce the presence of foreign particulates, kill contaminants, and remove loosely bound inhibitors. It was determined that the washing step did not significantly impact biomass solubilization assays (i.e., washed biomass only showed an extra 1 % better solubilization than unwashed *Populus* in our SHF controls—data not shown).

Carbohydrate content in fresh and post-fermentation biomass solids was determined by quantitative saccharification assay NREL/TP-510-42618 and HPLC method NREL/TP-510-42623. In short, biomass carbohydrates were acid hydrolyzed in 72 % w/w H_2_SO_4_ (0.1 g solids/mL acid) for 1 h at 30 °C, followed by further oligomer breakdown in 4 % w/w H_2_SO_4_ at 121 °C for 1 h. Samples were then neutralized to pH ~ 7 with calcium carbonate and 0.2 µm filtered before carbohydrate content (glucose, xylose, galactose, mannose, and arabinose) quantification by high performance liquid chromatography (LaChrom Elite™, Hitachi High Technologies America Inc.) against known standards. HPLC product separation was done using an Aminex™ HPX-87P column (Bio-Rad Laboratories Inc., Hercules, CA) at 0.6 mL/min flow rate of ultrapure water and column temperature of 80 °C; while signal was quantified on a refractive index detector (model L-2490) at 35 °C. Pentose and hexose degradation products (furfural and 5-hydroxymethyl furfural, respectively) were also quantified (UV–Vis L-2420 detector) to confirm optimum acid solubilization with only trace amounts of monomeric sugar breakdown. Total lignin content and the S/G ratio were determined by pyrolysis molecular beam mass spectrometry with methods previously described [40]. Total lignin content determinations in washed biomass which are normalized to gram solids may have underestimated the true values due to insufficiently dried biomass. We show that relative lignin content remained comparable between low S/G and high S/G biomasses.

### Consolidated bioprocessing (CBP): culturing conditions and fermentation product quantification

Media bottles for small-scale CBP screenings (at 50 mL culture volume) were prepared to anaerobic conditions by autoclave sterilization of solution A followed by vigorous sparging with nitrogen gas, followed by the sterile addition of all other components in aliquots from pre-filtered stocks. The final mixtures were sparged with nitrogen once again, for a minimum of 20 min. Inocula were prepared by starting an overnight culture in 2.5 g/L Avicel-MTC medium from glycerol-stocks and then transferring a 20 % v/v aliquot of growing culture to a fresh 2.5 g/L Avicel-MTC bottle for a second overnight growth phase. This process ensured a uniform and fast-growing inoculum source for all CBP-based experiments. All CBP-based experiments, in batch bottles or pH-controlled bioreactors were carried out in triplicate for each tested biomass substrate. The inoculation of bottles and bioreactors was always made with 10 % v/v freshly grown cultures.

### CBP conversion screening of initial four *Populus* phenotypes

*C. thermocellum* was grown in batch bottles with 5 g/L *Populus* biomass (dry basis) for 96 h at 60 °C with moderate shaking (150 rpm in MaxQ 6000, Thermo Scientific, Marietta, OH). At 24 h intervals, the bottles were vented with a syringe needle for 20 s inside an anaerobic cabinet (Coy Laboratories Products Inc., Grass Lake, MI) and the weight was recorded. The loss in weight due to release of fermentation gases was plotted over time and used as an indicator of overall bioconversion progress and has been used previously [41]. End-point aqueous samples were collected for fermentation product quantification by HPLC.

### CBP of low S/G and high S/G *Populus* in pH-controlled bioreactors

Microbial bioconversions were performed in 1 liter Sartorius BIOSTAT QPlus bioreactors (Sartorius Stedim Biotech, Göttingen, Germany), operated at 60 °C, pH 7 (titrated with 3 N KOH), and 200 rpm impeller mixing. Media were prepared in bioreactors at a final volume of 0.4 liters by autoclave sterilization of solution A (containing 20 g/L *Populus* biomass or 10 g Avicel), followed by cooling under continuous nitrogen sparging for 18 h. Medium components B, C, D, E, and F were then added to the reactors under sterile conditions and the pH probe was recalibrated. A mixture of 20 % CO_2_/80 % N_2_ gas was sparged through the reactors until the media pH reached 6; gas sparging was then switched to nitrogen for the duration of the experiment (at 0.4 mL/min mass flow rate) and the pH was raised and maintained to 7 with base addition before finally adding aliquots of fresh inoculum (at 10 % v/v). At regular intervals, solids-free aqueous samples were collected and analyzed for monomeric sugars and fermentation products by HPLC. Fermentation gases passed through a 4 °C cooled condenser and a water bubble trap where traces of fermented ethanol and organic acids were also detected and quantified at the end of experiment. Water trap ethanol accounted for up to 5 % of the total fermentation ethanol and was not included in further calculations (as reported in Fig. 3).

HPLC analysis of all hydrolysis and fermentation samples used a similar method to the carbohydrate quantification. Samples were 0.2 µm filtered and acidified to pH 2 with 2 M H_2_SO_4_ and the products were separated using an Aminex HPX-87H column (Bio-Rad Laboratories Inc., Hercules, CA) at 60 °C, at a flow rate of 0.5 mL/min with 5.0 mM H_2_SO_4_ mobile phase. Separated products were detected by refractive index (detector model L-2490 at 50 °C) and quantified against known standards.

Total solids solubilization at the end of CBP fermentations was calculated by determining the dry weight of residual solids. In short, 50 mL of well-mixed culture medium with biomass residues was collected and filtered through glass microfibre filters (Whatman GF/D, 47 mm diameter, GE Healthcare Life Sciences, Buckinghamshire, UK), then dried overnight at 45 °C.

### Determination of sugar release via free-enzyme hydrolysis and yeast-based ethanol production

Separate hydrolysis and fermentation (SHF) of low and high S/G *Populus* samples was done in batch bottles with a 5.0 % (w/v) solids loading in a 20 mL final volume. In the initial hydrolysis step, the biomass, enzymes, 0.05 mL streptomycin (0.063 mg/ml final concentration), and 16 mL of ultrapure water were mixed and incubated with gentle shaking at 50 °C for 5 days. Cellulase mix Cellic^®^ Ctec2 was loaded at 24 FPU/g cellulose, while the *β*-glucosidase Novozymes 188 and hemicellulases Cellic^®^ Htec2 were loaded at 25 and 20 % volume ratios to Ctec2. Novozymes 188 was purchased from Sigma Aldrich and other enzymes were donated by Novozymes North America (Franklinton, NC). Sugar-release aqueous samples were collected at the end of hydrolysis and the bottles inoculated with a mixture of exponential growth *Saccharomyces cerevisiae* D5A (ATCC 200062), citrate buffer (50 mM final concentration), and yeast extract (0.5 % w/v final concentration). Sugar fermentation continued at 35 °C for 3 days with mixing, and weight loss measurements were taken at regular intervals to monitor progress. Fermentation ethanol yield was determined at end-point by HPLC quantification with the same procedure described for CBP.

### Identification of potential biomass-derived inhibitors in the CBP fermentation metabolome

CBP fermentation samples of the low S/G and high S/G *Populus*, collected post-inoculation (0 h), during exponential growth (71 h) and at end-point (140 h), were analyzed for the presence of potential microbial inhibitors by gas chromatography-mass spectrometry (GC–MS) using previously described methods [32].

### Screening the inhibitory effect of *p*-hydroxybenzoic acid

Media batch bottles prepared with 20 g/L biomass of the high S/G *Populus* were supplemented with *p*-hydroxybenzoic acid at 4 and 40 mg/L final concentrations, inoculated with *C. thermocellum* cultures and incubated at 60 °C for 120 h. At regular intervals, aqueous aliquots were collected and analyzed by HPLC for fermentation products. Cultures without *p*-hydroxybenzoic acid additions and un-inoculated media controls were used as well.

### Lignin and cellulose characterization

Starting biomass and the recovered CBP post-fermentation solids were frozen at −80 °C and freeze-dried to remove moisture. Extractives were removed from the biomass via Soxhlet extraction with dichloromethane for 4.5 h under reflux and the extractive-free samples were air-dried overnight prior to further sample preparation.

### Molecular weight of lignin

The freeze-dried biomass was ball-milled to a fine powder at 520 rpm for 90 min. Samples of 300 mg were hydrolyzed in stoppered glass tubes with 20 mL of an enzyme cocktail containing cellulases from *Trichoderma sp.* (C1794, Sigma Aldrich, St. Louis, MO), cellulases from *Trichoderma reesei* ATCC 26921 (C2730, Sigma Aldrich, St. Louis, MO), and a *β*-glucosidase from almonds (G0395, Sigma Aldrich, St. Louis, MO) that had been pre-mixed in 20 mM sodium acetate buffer (pH 5.0). After incubation with shaking (200 rpm) at 37 °C for 48 h, the tubes were centrifuged at 8228×*g* (8000 rpm) for 10 min, and the supernatant was discarded. A new batch (20 mL) of the enzyme mixture was prepared and added to the pellet. Incubation resumed for an additional 48 h and the supernatant was discarded again. The pellet was washed twice with deionized water, frozen, and freeze-dried. In order to isolate lignin, *p*-dioxane (96 % v/v) was added to the dry pellet at 10 mL/g biomass and the mixture was stirred at room temperature for 24 h. The mixture was then centrifuged, the supernatant collected, and the remaining solids were treated with *p*-dioxane (96 % v/v) again under the same conditions. Supernatants from both extractions were combined, *p*-dioxane was evaporated using rotary evaporator, and freeze-dried to recover the isolated lignin. Lignin was derivatized by mixing 10 mg of isolated product with 1 mL of 1:1 anhydrous pyridine/acetic anhydride for 24 h at room temperature, and the reaction mixture was quenched with 5 mL ethanol. Derivatized lignin was washed several times with ethanol via rotary evaporation to remove residual chemicals and vacuum-dried. It was then dissolved in tetrahydrofuran (THF) (at 2 mg/mL), and filtered through 0.45 µm PTFE membrane. Molecular weight analysis was conducted by gel permeation chromatography (SECurity GPC 1200 System, Polymer Standards Services, Warwick, RI) containing four Waters Styragel columns (HR1, HR2, HR4, and HR6), an Agilent refractive index detector, an Agilent UV-detector (Agilent Technologies, Santa Clara, CA) with a THF mobile phase at 1 mL/min flow rate. The molecular weights of lignin were calculated against polystyrene standards in the 0.58 × 10^3^ to 5.38 × 10^5^ g/mol range.

### Degree of polymerization of α-cellulose

Freeze-dried biomass was delignified by treatment of 0.6 g samples with 5 % w/w peracetic acid for 24 h at 25 °C, then washed by water filtration several times and air-dried overnight. Hemicellulose was removed by solubilization in 17.5 % w/w NaOH for 2 h and 8.75 % w/w NaOH for an additional 2 h, and the insoluble cellulose was recovered and cleaned by filtration and washing with 1 % w/w acetic acid and ultrapure water several times. The cellulose was frozen, freeze-dried overnight, and vacuum-dried for an additional night. It was then derivatized using anhydrous pyridine (4 mL) and phenyl isocyanate (0.5 mL) over 3 days at 55 °C, and the reaction was quenched with methanol. The derivatized cellulose was regenerated in 7:3 methanol/water and then purified by repeat centrifugation at 8228×*g* (8000 rpm) for 10 min with resuspension in methanol/water and twice in water alone. The tricarbanilated cellulose was vacuum-dried overnight, then dissolved overnight in THF (1:1 mg/mL), and the solution was filtered using a 0.45-µm PTFE syringe filter. Cellulose molecular weight was determined by gel permeation chromatography against polystyrene standards, with a 1-mL/min flow rate of the THF mobile phase. The degree of polymerization (DP) was calculated as the fraction of the number-average molecular weight (*M*_n_) or the weight-average molecular weight (*M*_w_) to 519 g/mol.

### Cellulose crystallinity

Hemicellulose was hydrolyzed from 300 mg holocellulose (obtained through delignification, as described above) through reflux with 2.5 M HCl for 90 min and the recovered cellulose washed several times with ultrapure water by filtration. Cellulose crystallinity in moist samples was calculated from the attenuated total reflectance-Fourier transform infrared (ATR-FTIR) spectra obtained at 4 cm^−1^ resolution with 64 scans on the PerkinElmer Spectrum 100 FTIR spectrometer (PerkinElmer, Houston, TX) equipped with a universal crystal ATR accessory [42].

In conjunction with ATR-FTIR analysis, cellulose crystallinity was determined with ^13^C cross-polarization magic-angle spinning (CP-MAS) NMR spectroscopy. The acid-hydrolyzed cellulose was packed into 4 mm cylindrical ceramic MAS rotors analyzed on the Bruker DSX-400 NMR spectrometer (Bruker, Billerica, MA, United States) at an operating frequency of 100.55 MHz in a Bruker double-resonance MAS probe head. The CP-MAS experiments were conducted with the following parameters: 2024 scans, 1.5 ms contact pulse, 4 s recycle delay, and a 5 µs (90°) pulse. Additionally, each sample was spun at 8 kHz [43].

In the resulting spectra, C4 in cellulose is split into two peaks that correspond to the crystalline and amorphous character of cellulose. The “amorphous” peak lies between 80 and 86 ppm, while the “cellulose” peak lies between 86 and 92 ppm. The crystallinity index (CrI) of cellulose can be calculated from the areas of the peaks between 80–86 ppm (*A*_80–86 ppm_) and 86–92 ppm (*A*_86–92 ppm_), as indicated in Eq. 1.

1 $${\text{CrI}} \,\left( \% \right) = \frac{{A_{{86 - 92\,{\text{ppm}}}} }}{{A_{{80-86\,{\text{ppm}}}} + A_{{86-92\,{\text{ppm}}}} }} \times 100.$$

### Cellulose accessibility by Simons’ staining

Direct Blue 1 (Pontamine Fast Sky Blue 6BX) and Direct Orange 15 (Pontamine Fast Orange 6RN) were obtained from Pylam Products Co. Inc. (Garden City, NY) and used at a working concentration of 10 mg/mL in water. For Direct Orange 15, the low molecular weight components were first removed by ultrafiltration through 100 K membranes with ~200 kPa nitrogen gas (Amicon stirred cell, EMD Millipore Corp., Billerica, MA)—as this fraction demonstrated similar cellulose affinity with Direct Blue 1 [44]. The residual weight of filter-trapped dye was used to estimate the effective Direct Orange concentration in the filtrate and the stain was then further diluted to the working concentration. The dye mix adsorption isotherm was determined using a series of 1:1 mixtures with increasing concentrations.

Fresh and post-fermentation *Populus* samples (~10 mg) were suspended in 0.1 mL buffered saline solution (0.3 M Na_3_PO_4_ and 1.4 mM NaCl at pH 6) and the volume was adjusted to 1.0 ml with deionized water. The samples were incubated with shaking at 70 °C for 6 h, then centrifuged at 10,252×*g* (10,000 rpm) for 5 min, and the supernatant was collected. Supernatant absorbance was measured using the Lambda 35 UV–Vis Spectrophotometer (PerkinElmer, Waltham, MA), and the calculations for analysis were adapted from Chandra et al. [45]

### Statistical analysis

The modified *z*-score, proposed by Iglewicz and Hoaglin, was used to detect outliers in this study due to its relevancy to small sample sizes. The modified *z*-score (*M*_i_) was calculated for each datum using Eq. 2. Any datum possessing a modified *z*-score ≥3.5 was labeled as a potential outlier and removed from further analysis.

2 $$M_{\text{i}} = \frac{{0.6745\left( {x_{\text{i}} - \tilde{x}} \right)}}{\text{MAD}},\quad {\text{where}}\,\,{\text{MAD}} = {\text{median}}\left( {\left| {Y_{\text{i}} - \tilde{Y}} \right|} \right),$$

where appropriate, samples means were compared using a two-sample *t* test and the *p* value was calculated with equal or different variances based on the results of a previously run *F* test. Origin Pro software was used for the calculations, and the *p* value represented the probability that observed differences between means was due to random chance (i.e., when *p* < 0.05 the probability for random chance is very small, and the means are considered statistically different).

## Additional file

10.1186/s13068-016-0445-x Carbohydrate composition of initially screened *Populus* biomass. **Figure A.2.** Fermentation products of Avicel-control CBP cultures. **Figure A.3.** Carbohydrate content in *Populus* before and after repeat autoclave sterilization. **Figure A.4.** Lignin content in *Populus* before and after repeat autoclave sterilization.

## Abbreviations

ATR-FTIR: attenuated total reflectance-Fourier transform infrared
CBP: consolidated bioprocessing
DP: degree of polymerization
FPU: filter paper units
GC–MS: gas chromatography–mass spectrometry
GPS: gel permeation chromatography
HPLC: high pressure liquid chromatography
*M*_w_: weight-average molecular weight
*M*_n_: number-average molecular weight
S/G: syringyl-to-guaiacyl ratio
SHF: separate hydrolysis and fermentation
