# Supplementary material for: Consolidated bioprocessing of Populus using Clostridium (Ruminiclostridium) thermocellum: a case study on the impact of lignin composition and structure
Source: Biotechnol Biofuels. 2016 Feb 4;9:31. doi: 10.1186/s13068-016-0445-x (PMC4743434; doi:10.1186/s13068-016-0445-x)
Supplement: Supplementary file 1 — 10.1186/s13068-016-0445-x Carbohydrate composition of initially screened Populus biomass. Figure A.2. Fermentation products of Avicel-control CBP cultures. Figure A.3. Carbohydrate content in Populus before and after repeat autoclave sterilization. Figure A.4. Lignin content in Populus before and after repeat autoclave sterilization. [file 13068_2016_445_MOESM1_ESM.pdf]

Additional file 1

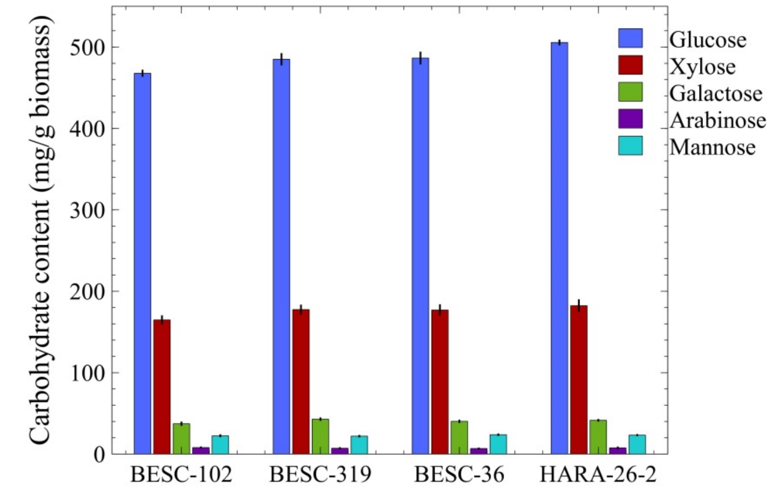

	BESC-102	BESC-319	BESC-36	HARA-26-2
Total lignin	24.9%	24.4%	24.8%	23.7%
S/G ratio	1.2	2.0	2.0	2.1

Figure A. 1. Selected *Populus* with comparable carbohydrate and total lignin content; three individuals had similar S/G ratios while a forth had the lowest possible S/G ratio composition

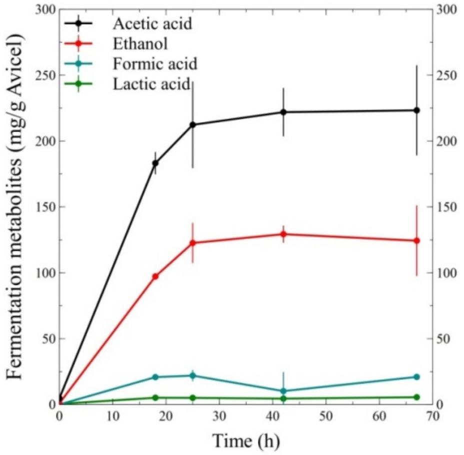

Figure A. 2. Control consolidated bioprocessing of 10g/L glucan (Avicel) showed near complete solubilization within 24 hours and normal fermentation metabolite output

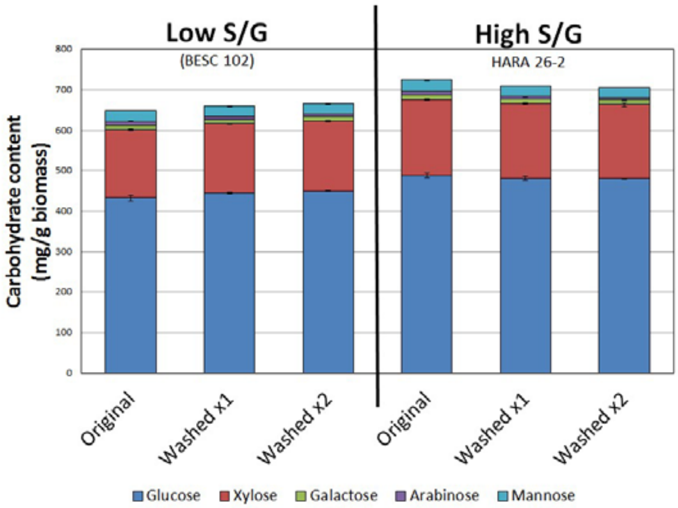

Figure A. 3. Carbohydrate content does not change significantly in *Populus* biomass after one or two wash cleaning cycles

	Total lignin (%)	S/G ratio
**Low S/G**		
Original	25	1.1
Washed x1	19	1.2
Washed x2	18	1.2
**High S/G**		
Original	24	1.9
Washed x1	17	2.1
Washed x2	16	2.1

Figure A. 4. Total lignin content decreases after the first wash cycle and no significant change with subsequent washes; the lignin S/G ratio increases very slightly in both *Populus* samples
